# Interaction of Caffeine with Model Lipid Membranes

**DOI:** 10.1021/acs.jpcb.1c04360

**Published:** 2021-09-01

**Authors:** Letizia Tavagnacco, Giacomo Corucci, Yuri Gerelli

**Affiliations:** † CNR-ISC and Department of Physics, Sapienza University of Rome, Piazzale A. Moro 2, 00185 Rome, Italy; ‡ Institut Laue-Langevin, 71 avenue des Martyrs, 38000 Grenoble, France; § Department of Life and Environmental Sciences, Marche Polytechnic University, Via Brecce Bianche, 60121 Ancona, Italy

## Abstract

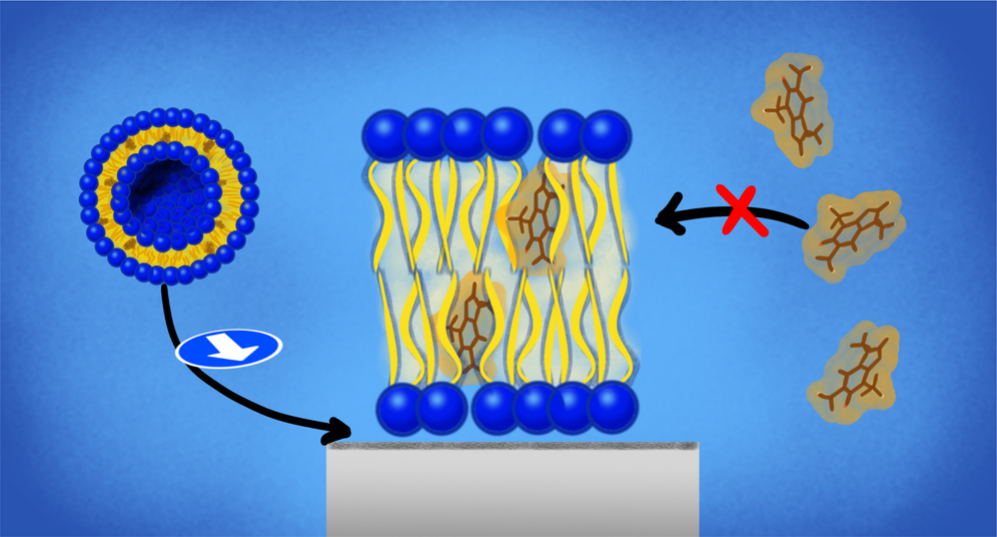

Caffeine is not only a widely consumed active stimulant, but it is also a model molecule commonly used in pharmaceutical sciences. In this work, by performing quartz–crystal microbalance and neutron reflectometry experiments we investigate the interaction of caffeine molecules with a model lipid membrane. We determined that caffeine molecules are not able to spontaneously partition from an aqueous environment, enriched in caffeine, into a bilayer. Caffeine could be however included in solid-supported lipid bilayers if present with lipids during self-assembly. In this case, thanks to surface-sensitive techniques, we determined that caffeine molecules are preferentially located in the hydrophobic region of the membrane. These results are highly relevant for the development of new drug delivery vectors, as well as for a deeper understanding of the membrane permeation role of purine molecules.

## Introduction

Caffeine is an aromatic compound, usually found in coffee and tea, which belongs to the class of purine molecules. As such, its molecular structure consists of a flat heteroatomic bicyclic ring, which brings hydrophobic properties and limited solubility in water, although it can be increased by heating the solution.^1^ It is known that purine systems are characterized by the vertical stacking of their planar bases.^2^ Hydrophobic association, the interaction of amphiphilic systems mediated by water, which is fundamental in many different phenomena,^3,4^ is also at the origin of caffeine vertical stacking.^5,6^ Using small-angle neutron scattering and molecular dynamics simulations, it was shown that in a 0.1 mol/kg solution of caffeine at room temperature, individual caffeine molecules are in equilibrium with dimers and trimers, mostly.^7^ This natural coexistence is crucial for the investigation of the interaction of purine bases in DNA,^8,9^ and purine derivatives with other biomolecules, such as sugars,^10−12^ neurotransmitters,^13^ adenosine triphosphate,^14^ amyloid peptides,^15^ and phospholipids.

Interaction of purine molecules with cell membranes is of large interest for making predictions for the pharmacokinetic properties of new synthetic purine drugs,^16^ as mercaptopurines, and for the large use of caffeine as coadjuvant of drug molecules,^17^ especially of analgesics.^18,19^ In pharmaceutical sciences, caffeine is commonly used as a psychoactive drug, and it has been also proposed for the treatment of cellulitis.^20−23^ In this case, to enhance its delivery via skin permeation, caffeine was encapsulated in liposomes having lecithin as the main component plus a variable class of excipients. Several works have reported that caffeine has a low affinity to liposomes composed only of lecithin (mainly phosphatidylcholine-based lipids), the encapsulation efficiency being very low^20,22−24^ and caffeine being spontaneously released from these lipid carriers. The use of excipients increased the encapsulation efficiency and delayed the spontaneous caffeine release. In addition to delivery via skin permeation, whether used as an adjuvant or as a drug itself, caffeine molecules have to cross cell membranes to be properly delivered within the human body.

To our knowledge, despite these studies on the encapsulation in liposomes, there are very few results in literature providing a clear description of the mechanism of interaction between caffeine and the lipid components of cell membranes.^25,26^ The information becomes even more limited if we consider investigations performed at molecular or atomic length scales.^27,28^ Paloncýová *et al*.^27^ performed molecular dynamics (MD) simulations and quantum--chemical calculations combined with statistical thermodynamics (using COSMOmic^29^) to determine the partitioning of several xenobiotic compounds into lipid bilayers by calculating the free-energy profiles normal to the membrane surface. In the case of caffeine, the authors reported it can penetrate into a model biomembrane from an aqueous environment and position below the phospholipid headgroup without protruding deeper into the hydrophobic portion of the bilayer. MD simulations showed that the adsorption of caffeine from an aqueous environment into a bilayer is energetically favored. On the other hand, the free-energy profile obtained with COSMOmic was characterized by a minimum locating caffeine molecules in the proximity of lipid headgroups but in an aqueous environment.^27^ These two contradictory results might be a signature of nonspecific interactions between caffeine and a phospholipid membrane. These results might also support the low encapsulation efficiency characteristic of pure lecithin liposomes and their spontaneous tendency of releasing caffeine molecules.^20,22,23^ All of the studies of encapsulation efficiency were performed dissolving together caffeine and phospholipids in organic solvents before the preparation of liposomes. Even if the encapsulation efficiency resulted to be limited, between 3 and 9% of the total caffeine content resulted enclosed in the bilayer part of the liposomes. After 2 and 12 h, this content decreased by 23 and 57%, respectively.^20^ The remaining caffeine molecules were found to be trapped within the lipid bilayer for the explored timescales. Since these investigations were performed to evaluate the efficiency of liposomal formulations, the authors did not provide information on the location of caffeine molecules within the bilayer. In the recent work of Khondker *et al.*,^28^ such a characterization was performed experimentally by means of X-ray diffraction (XRD) and computationally by means of MD simulations. The samples consisted of highly oriented, multilamellar membrane stacks obtained upon evaporation of a chloroform solution in which 1-palmitoyl-2-oleoyl-*sn*-glycero-3-phosphocholine (POPC) phospholipids were dissolved together with caffeine (3 mol % concentration). After their formation, the samples were kept and measured in humid air. For this reason, caffeine could not be released from the membrane stacks. MD simulations were performed on a single bilayer system with a lipid/caffeine ratio equal to that of the samples measured by XRD. The authors described, at the molecular level, the structural modifications induced by the presence of caffeine molecules in POPC bilayers. Both XRD and MD simulations located caffeine molecules at the level of the phosphate group, thus confirming earlier predictions.^27^ Spontaneous partitioning of caffeine into a POPC bilayer was observed only through MD simulations since the samples for XRD were preloaded with caffeine and not in contact with an aqueous environment. The spontaneous, nonspecific, partitioning of caffeine into a bilayer composed only of zwitterionic phospholipids is in contradiction with the experimental evidence of spontaneous release and low encapsulation efficiency of lecithin liposomes in solution.

In this work, we are bridging this gap by measuring, in real-time, the absence of partitioning of caffeine molecules from an aqueous environment into a supported lipid bilayer (SLB) by means of a quartz–crystal microbalance with dissipation monitoring (QCM-D) and neutron reflectometry (NR) techniques. The structure of SLBs preloaded with caffeine was also characterized at the molecular level. Our results describe a scenario in which caffeine cannot be readily exchanged, in both directions, between a phospholipid SLB and the aqueous environment. Because of their large hydrophobic surface, caffeine molecules remain within the hydrophobic portion of the bilayer, if premixed with phospholipids before the self-assembly process. On the contrary, despite their large hydrophobic surface, caffeine molecules do not partition into a bilayer environment from a water solution. This last experimental observation can be a consequence of the self-stacking behavior typical of purine systems in water that was not taken into account in previous computer simulations, where only an individual caffeine molecule was considered.

## Methods

### Materials

1-Palmitoyl-2-oleoyl-*sn*-glycero-3-phosphocholine (POPC) was purchased from Avanti Polar Lipids (Alabaster). Caffeine and chloroform were purchased from Sigma-Aldrich (Saint-Quentin Fallavier, France). For caffeine-free samples, lipid stock solutions were prepared by dissolving phospholipids in chloroform. Vesicles preloaded with caffeine were prepared by dissolving 0.14 mg of caffeine together with 10 mg of phospholipids in chloroform leading to a caffeine concentration of 5 mol %, a value similar to that used in other studies.^26,28^ In both cases, chloroform was evaporated under a nitrogen flow to form a dry film on the walls of a glass vial. Any remaining chloroform was removed by placing the sample under vacuum overnight. The films were treated with five cycles of freezing in liquid nitrogen and thawing at 40 °C using a water bath. The films were then rehydrated in either H_2_O (Milli-Q water) or D_2_O (Euroisotopes, France) to a final concentration of 1 mg/ml. The vesicles were obtained by tip sonication (Bandelin Sonopuls) for 30 min with 50% maximum amplitude, with a 2 s pulse-on and 3 s pulse-off sequence. This protocol allowed us to obtain a clear vesicle suspension. The samples in which *d*_31_POPC (1-palmitoyl-*d*31-2-oleoyl-*sn*-glycero-3-phosphocholine, purchased from Avanti Polar Lipids) replaced POPC were prepared following the same protocol.

### Vesicle Fusion Protocol

**Figure 1:**
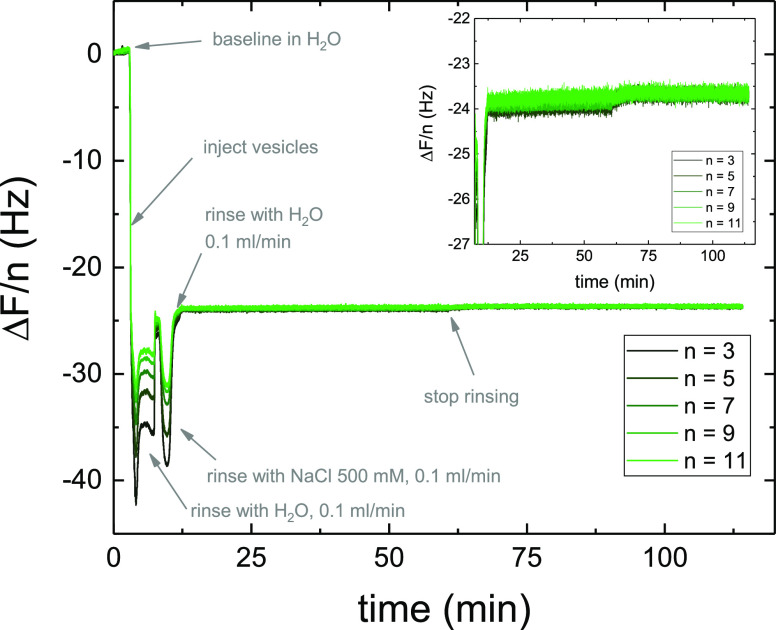
Normalized frequency shifts Δ*F*/*n* (overtones from 3 to 11) as a function of time measured during and after the formation of a SLB from POPC vesicles preloaded with caffeine. The first rinsing, performed in H_2_O, did not facilitate vesicle fusion while the osmotic shock resulted to be more efficient. The SLB was formed at *t* = 12.5 min. Rinsing by flowing H_2_O at 0.1 mL/min was then performed until *t* = 62.5 min. The perfect overlap of all of the overtones indicates the formation of a rigid thin film and therefore the validity of the Sauerbrey equation. Using eq 1, Δ*m* resulted in 1.36 ng/cm^2^. A zoom of the curve for the long-term behavior is given in the inset.

For both QCM-D and NR techniques, lipid bilayers were prepared by exploiting the vesicle fusion method.^30^ The vesicle fusion protocol was optimized by means of QCM-D experiments. Vesicle solutions were injected into the sample cell and left to equilibrate for 10 min. A salt solution (500 mM NaCl) was then injected into the cell to promote vesicle fusion via osmotic shock. This step was necessary since a simple rinsing step with H_2_O did not promote the formation of a SLB as shown in Figure 1. The cell was finally rinsed by flowing H_2_O to remove any unbound material.

### Quartz–Crystal Microbalance with Dissipation Monitoring

QCM-D measurements were performed to monitor the interaction of free caffeine in solution with SLBs composed of POPC deposited on the surface of the QCM-D sensors and to optimize the vesicle fusion protocols for vesicles preloaded with caffeine. All measurements were performed on an E4 apparatus (Q-Sense, Sweden) equipped with four thermally insulated flow modules. Silicon dioxide coated quartz sensors were used as supporting surfaces (Biolin Scientific, Sweden). Prior to usage, they were cleaned in a 2% neutracon (Decon Laboratories, U.K.) solution in a sonication bath, rinsed, dried, and exposed to air plasma for 2 min (Harrick Plasma). SLBs were deposited via vesicle fusion after the acquisition of a stable (±0.5 Hz) baseline in water. During the entire duration of the experiment, frequency and dissipation data were acquired every 2 s. All measurements were replicated four times to ensure reproducibility.

#### Caffeine-Free SLBs

Experiments on caffeine-free SLBs were carried out by exposing SLBs to a 0.1 mol/kg caffeine solution (0.1 mol/kg = 0.1 *m* is the solubility limit at 298 K^31^). The temperature was kept constant at 28 ± 0.1 °C. After caffeine injection, incubation was monitored by acquiring data for a minimum of 1 h and a maximum of 16 h. At the end of incubation, the samples were rinsed by flowing H_2_O at 0.1 mL/min to compare the signals at the beginning and at the end of the experiment.

#### SLBs Preloaded with Caffeine

Measurements on SLBs preloaded with caffeine were performed fusing vesicles containing caffeine onto the sensor surface. After the formation of a SLB, the cell was rinsed by flowing H_2_O (0.1 mL/min) to remove any unbound material. Frequency and dissipation signals were acquired for 120 min recording data every 2 s.

#### QCM-D Data Analysis

Since all of the systems, at equilibrium, were characterized by very small dissipation values and by overlapping normalized frequency shifts, data analysis was performed using the Sauerbrey equation (eq 1) that relates a frequency shift Δ*F* to a change in adsorbed mass Δ*m*.^32,33^ In its simplified form, the Sauerbrey equation can be written as
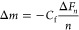
1where *C*_f_ is the mass sensitivity constant (*C* = 17.7 ng/(cm^2^ Hz) for a sensor having a 5 MHz fundamental frequency), *n* is the overtone number, and Δ*F*_*n*_ is the change in frequency of the *n*th overtone. As a reference, for a high coverage phospholipid bilayer, normalized frequency shifts are expected to be nearly −25 Hz for a 5 MHz sensor.^34^

### Neutron Reflectometry

NR experiments were performed using silicon single crystals as solid substrates (8 × 5 cm^2^ surface, 1.5 cm thick, cut along the 111 plane, polished with 3 Å RMS roughness). They were cleaned by washing with chloroform, acetone, and ethanol. Ultrasound was used to improve the cleaning efficiency of the solvents. The substrates were kept in each solvent for 15 min, rinsed with Milli-Q grade water, and dried before being immersed in the next solvent. To make the surfaces hydrophilic and to remove any remaining trace of organic molecules, they were exposed to air plasma for 2 min. The substrates were then enclosed into solid/liquid cells (provided by the Intitut Laue-Langevin, France). Cells, prefilled with H_2_O, were equipped with inlet and outlet valves, allowing the exchange of the water subphase (using a high-performance liquid chromatography setup) and the manual injection of vesicle and caffeine solutions.

NR measurements were performed on the D17 reflectometer^35^ (Institut Laue-Langevin, France) in time-of-flight mode using wavelengths from 2 to 20 Å and two angular configurations, θ = 0.8 and 3.0°, covering a *Q*-range from 0.008 to 0.28 Å^–1^. *Q* indicates here the component of the exchanged wave-vector along the direction normal to the sample surface.^36^ Reflectivity curves, *R*(*Q*), were obtained from the ratio between the intensity of the reflected (specular) and incident neutron beams. *R*(*Q*) curves were measured for (i) bare silicon substrates, (ii) SLBs without caffeine, (iii) SLBs preloaded with caffeine, and (iv) pristine SLBs exposed to a caffeine bulk solution. To increase the accuracy of the modeling, reflectivity curves were collected by exposing the samples to aqueous environments characterized by different D_2_O/H_2_O ratios. This method is known as contrast variation.^37^ In particular, 100% D_2_O, 100% H_2_O, silicon-matched water (SiMW, a 38:62 D_2_O/H_2_O mixture by vol), and four-matched water (4MW, a 66:34 D_2_O/H_2_O mixture by vol) were used. The scattering length density (SLD) of SiMW equals the one of crystalline silicon (2.07 × 10^–6^ Å^–2^) while SLD is 6.35 × 10^–6^ Å^–2^ for D_2_O, 4.0 × 10^–6^ Å^–2^ for 4MW, and −0.56 × 10^–6^ Å^–2^ for H_2_O. As in the case of QCM-D experiments, all measurements were performed at 28 °C. *R*(*Q*) curves referring to the same sample and collected upon contrast variation were analyzed simultaneously using the Aurore software application.^38^

The result of NR data modeling is a set of scattering length density profiles describing the distribution (or volume fraction) of nuclei along the perpendicular direction with respect to the supporting interface.^39−43^ This information relates to the distribution of chemical species in the sample.

#### Modeling of NR Data

NR data were modeled by representing the SLD profile of a sample as a finite series of slabs. By going from the silicon substrate to the water phase, both described as infinitely thick media, the model included slabs describing (i) the native silicon oxide layer, (ii) a water gap between the oxide and the bilayer, (iii) inner headgroups, (iv, v) two hydrophobic regions, and (vi) outer headgroups. Each slab in the model was characterized by a group of four parameters, namely, thickness, SLD of the dry components, water volume fraction, and interfacial roughness. The latter corresponds to the width of an error function curve. The NR data for bare silicon substrates were analyzed, which included only the crystalline silicon, the oxide layer, and the water subphase in the model; the resulting parameters were then kept fixed during the analysis of the samples deposited on top of each individual substrate. The modeling of caffeine-free SLBs was performed using molecular constraints linking the parameters of the tail region to that of the headgroup. These constraints include mass conservation and a link between volume, thickness, and molecular area of the tail and headgroup regions. A detailed description of these constraints is given in the Supporting Information material (Section 1.1). In the case of caffeine-containing SLBs, these molecular constraints could not be applied because of the expected modification of phospholipid packing and SLD values. In this case, to keep the number of free parameters limited, the values derived from the analysis of pristine POPC SLBs were used as a starting point for the analysis under the hypothesis that caffeine molecules would induce only a small perturbation to the bilayer structure. From the set of parameters describing the SLD profile, reflectivity was calculated using the Parratt formalism^44^ within the Aurore software application.^38^ The *R*(*Q*) model curves were fitted against the experimental data to obtain the best set of optimized parameters for each sample and, in turn, the corresponding best SLD profiles. As already mentioned, the reflectivity data collected in different contrasts for the same sample were co-refined. Errors on parameters were calculated using the MINOS routine, evaluating the increase in the χ^2^ value caused by changes in the optimized parameter values, provided by the MINUIT^45^ package.

## Results

### SLBs Preloaded with Caffeine

To validate the possibility of forming SLBs with very large surface coverage starting from POPC vesicles containing caffeine, we first performed QCM-D experiments (Figure 1). The Δ*F*/*n* values overlapping at −24 Hz are indicative of the formation of a good-quality SLB mostly composed of POPC lipids.^34^ Moreover, upon extensive rinsing, the frequency shifts remained stable, as shown in Figure 1 (inset). The small change observed at *t* ∼ 62 min corresponds to the end of the rinsing step (when H_2_O flow was stopped). The stability of the signal over such a long time interval indicates that caffeine, if present, was not released from the SLB. However, since the normalized frequencies did not differ enough from those of a pure POPC SLB and the concentration of caffeine in the vesicles could be lower than the nominal one,^20,22,23^ it was not possible to measure the lipid/caffeine ratio in the SLBs.

**Figure 2:**
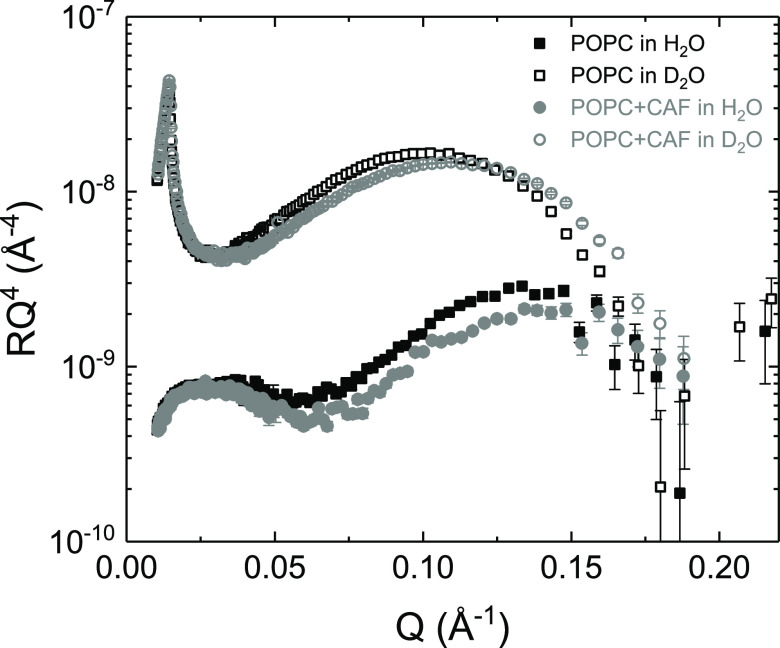
NR data for a POPC SLB (black symbols) and for a SLB formed from vesicles preloaded with caffeine (5 mol %, gray symbols). Open symbols: data in D_2_O; solid symbols: data in H_2_O.

**Figure 3:**
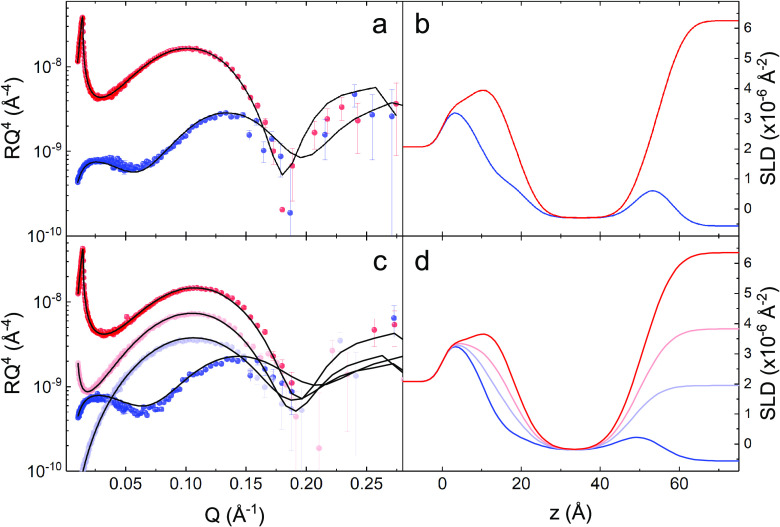
(a) NR curves (symbols) for a POPC SLB in D_2_O (red) and in H_2_O (blue). Solid lines represent the best model obtained by a global fit using the parameters reported in Table 1. (b) SLD profiles corresponding to the model fits reported in panel (a) (D_2_O (red) and H_2_O (blue)). (c) NR curves (symbols) for the SLB containing POPC and caffeine (at 5 mol %) in D_2_O (red), 4 MW (light red), SiMW (light blue), and H_2_O (blue). Solid lines represent the best model obtained by a global fit using the parameters reported in Table 1. (d) SLD profiles corresponding to the model fits reported in panel (c) (D_2_O (red), 4 MW (light red), SiMW (light blue), and H_2_O (blue)).

To determine quantitatively the lipid/caffeine ratio in the SLBs, NR experiments were performed. In Figure 2, the NR data for a sample composed only of POPC and one composed of POPC and caffeine are compared. This comparison clearly reveals that the presence of caffeine altered the overall bilayer organization. To avoid visual artifacts induced by the features of the silicon oxide layer, these two data sets were collected on the same exact solid substrate. Therefore, differences originated only from the presence of caffeine. The caffeine-containing sample was characterized by a lower reflectivity, in the mid-Q-range, for both D_2_O and H_2_O contrasts, indicating a change in composition. In the presence of caffeine, the main minimum in *R*(*Q*) *Q*^4^ is located at a larger *Q* value for the D_2_O contrast, indicating a lower total bilayer thickness. To better interpret these changes, all NR data were analyzed assuming a multilayer model commonly used for SLBs^38,46^ based on a bilayer divided into two leaflets, which, in turn, are split into hydrophilic (headgroup) and hydrophobic (tails) regions. Through the modeling, the thickness and composition of these regions were determined in terms of the SLD profiles. The NR curves for a pristine POPC bilayer and for a bilayer deposited from vesicles prepared in the presence of 5 mol % of caffeine are shown in Figure 3 (a and c, respectively). These curves were measured in D_2_O and H_2_O for the POPC bilayer while two additional contrasts^37^ were used for the caffeine-containing sample (namely, 4MW and SiMW). The NR data, fits, and SLD profiles for *d*_31_POPC SLBs, measured in the same conditions, are shown in the Supporting Information (Figure S1). The modeling of pristine SLBs returned parameters in full agreement with those already published by us^47^ and other groups.^48−51^ The full list of the structural parameters is given in Table 1. It is worth mentioning that at *T* = 28 °C, typical SLD values for the main components are 1.79 for the PC headgroups, −0.29 for the PO tails, and 3.3 for a caffeine molecule (all in 10^–6^ Å^–2^ units). The SLD values for phospholipids were calculated by assuming a lipid volume of 1256 ± 1 Å^3^, a dry headgroup volume of 332 ± 2 Å^3^, and, in turn, a volume of 923 ± 6 Å^3^ for the PO tails.^48,52^ The SLD value for caffeine was calculated assuming a molecular volume of 194 Å^3^.^53^ These values do not account for the contribution of hydration water. For SLBs preloaded with caffeine, the SLD values obtained for the different regions indicated that caffeine molecules are located in the hydrophobic portion of the bilayer. In fact, the SLD value of the tails increased from −0.29 × 10^–6^ to −0.25 × 10^–6^ Å^–2^ in the presence of caffeine, while the SLD value of the headgroup region remained unchanged. From the parameters reported in Table 1, the caffeine relative concentration in the SLB resulted to be 5.1 ± 0.5 mol % (18–19 lipid molecules per caffeine molecule), which is exactly the original concentration used to prepare this sample. The corresponding volume fraction of caffeine is 1.0 ± 0.1% of the volume of the hydrophobic SLB core or, alternatively, 0.8% of the total SLB volume. Alternative models, in which caffeine molecules were located in different regions of the SLBs or even absent, are discussed in Section 1.3 of the Supporting Information material. For samples composed of partially deuterated lipids, no changes in the tail region SLD were observed. This was expected since partially deuterated tails are characterized by a SLD value of 3.16 × 10^–6^ Å^–2^, which is very close to that of caffeine that was therefore contrast matched (i.e., invisible). Despite this lack of signal, these measurements ruled out the possibility to find caffeine molecules in the headgroup region, confirming the results obtained on protiated species. All measurements reveal a modification in the organization of the headgroups in the presence of caffeine; the amount of hydration water present in this hydrophilic region increased for all of the samples but remained negligible for the hydrophobic region. The total bilayer thickness decreased upon incorporation of caffeine, being *d*_*B*_ = 43.6 ± 0.8 Å for the POPC bilayer and 39 ± 1 Å for the preloaded SLB (see Table 1; *d*_B_ = 2(*t*_H_ + *t*_C_)). From the inspection of the structural parameters, this change in thickness originated from both hydrophilic and hydrophobic regions of the bilayer. This change is visible in Figure S3 in the Supporting Information, where SLD profiles for the POPC SLB and for the POPC + CAF SLB are displayed together.

**Table 1:** Structural Parameters Obtained from the Modeling of NR Data Collected on Protiated POPC Lipids (with and without Caffeine) Shown in Figure 3^a^^b^

parameters	POPC	POPC + CAF (5 mol %)
*t*_H_ (Å)	6.8 ± 0.2	5.3 ± 0.2
*t*_C_ (Å)	15.0 ± 0.2	14.4 ± 0.4
ρ_H_ (× 10^–6^ Å^–2^)	1.79	1.8 ± 0.1
ρ_C_ (× 10^–6^ Å^–2^)	–0.29	–0.25 ± 0.05
*f*_H_ (0:1)	0.18 ± 0.1	0.26 ± 0.08
*f*_C_ (0:1)	0	0.00 ± 0.01
σ (Å)	4.2 ± 0.3	5.7 ± 0.4

a For all samples, the two leaflets shared a common set of parameters (bilayers symmetric with respect to the center of the hydrophobic region). Parameters without error were kept fixed during the analysis.

b ρ_H_, *t*_H_, and *f*_H_ ⇒ SLD, thickness and water volume fraction of the headgroup regions; ρ_C_, *t*_C_, and *f*_C_ ⇒ SLD, thickness and water volume fraction of the tail regions; σ ⇒ interfacial roughness, common to all of the interfaces in the model; accuracy on ρ_H_ and ρ_C_ for POPC are, respectively, ±0.001 and ±0.002 in × 10^–6^ Å^–2^ units as determined by the accuracy on the molecular volume values.^48,52^

### Caffeine-Free SLBs

**Figure 4:**
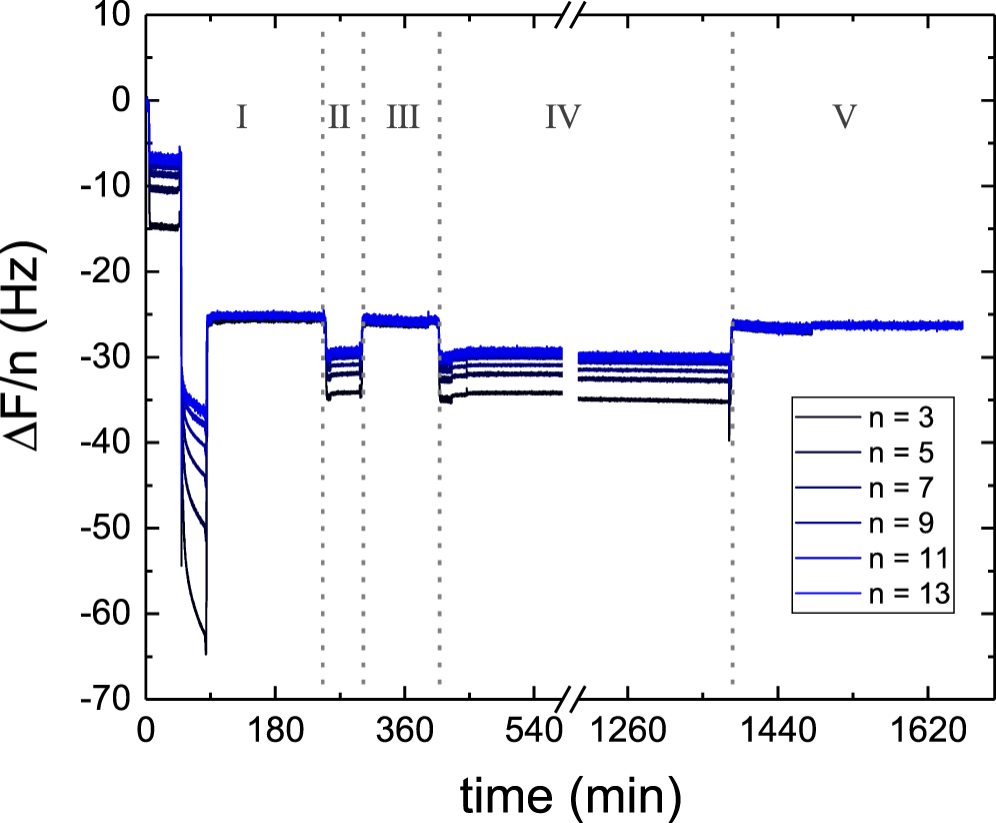
Normalized frequency shifts Δ*F*/*n* (overtones from 3 to 13) as a function of time measured during (I) the formation of a SLB, (II) 100 min incubation with 0.1 mol/kg caffeine solution, (III) rinsing with H_2_O, (IV) 16 h incubation with 0.1 mol/kg caffeine solution, and (V) final rinsing with H_2_O.

To determine if spontaneous partitioning of caffeine molecules from the aqueous solution into a bilayer environment can take place, we investigated a POPC bilayer exposed to a 0.1 mol/kg caffeine solution (0.1 mol/kg is the solubility limit at 298 K^31^). Figure 4 shows the QCM-D data for this experiment. When the caffeine solution was present in the sample cell, all of the frequencies decreased and split. Caffeine incubation, in stopped-flow conditions, was monitored for 100 min as well as for 16 h. In both cases, all frequencies went back to their original value upon rinsing. This behavior could be indicative of a transient nonspecific interaction between caffeine and POPC, but it is more likely to be a signature of a change in the viscosity of the solution due to the presence of caffeine at high concentrations. The same experiment was repeated using NR to determine if any process of adsorption or adhesion occurred. For this purpose, a POPC SLB was characterized in D_2_O and H_2_O before the incubation with caffeine. It was then exposed to a 0.1 mol/kg solution of caffeine in D_2_O. During the incubation, time-resolved measurements were performed acquiring the NR data every 5 min for a total duration of 1 h. These measurements were performed on a restricted Q-range (Figure S2) and using an instrumental configuration that is described elsewhere.^46^ At the end of the incubation period, the system was characterized, on the full Q-range, in D_2_O before rinsing, and then in D_2_O and SiMW after rinsing. In all of the steps (see Figure S2), no changes were observed, supporting the conclusion that frequency variations in QCM-D data were induced by a viscosity change and not by caffeine adsorption/adhesion. Even more importantly, these data clearly show that spontaneous partitioning of caffeine from solution to a bilayer environment is not likely to occur.

## Discussion

In earlier studies, the interaction of caffeine and lipid bilayers was mainly investigated with a focus on the encapsulation efficiency of liposome-based formulations without providing information, at the molecular level, on the positioning of the encapsulated caffeine molecules with respect to the bilayer. This question was addressed first by computer simulations^27^ and more recently by a combination of computer simulations and experiments.^28^ Numerical simulations found an affinity for the caffeine molecules to the headgroup-tail boundary region, i.e., the position occupied by the carbonyl-glycerol in a POPC bilayer.^54^ This positioning was further confirmed by experiments performed by means of XRD on multi-bilayer stacks in a humid environment. Computational methods also showed a tendency of caffeine molecules to partition spontaneously from a water solution into a bilayer structure.^28^ Our experiments, performed on single phospholipid bilayers in contact with an aqueous environment indicated that caffeine can only be present in a SLB if dissolved together with phospholipids, in an organic solvent, prior to the formation of the bilayer. By putting in contact an SLB with a caffeine solution at a concentration very close to the caffeine solubility limit, no partitioning of caffeine was observed by NR nor QCM-D for time intervals spanning over 16 h. Therefore, our findings support the idea that caffeine affinity for a bilayer environment is lower than that for an aqueous environment. This experimental observation might be related to the previous findings on caffeine self-stacking in solution.^6,7^ Caffeine partitioning was predicted from simulations in which a single caffeine molecule was present, a condition neglecting the influence of caffeine stacking on the nonspecific interactions with a lipid membrane. It is worth remarking that caffeine stacking was observed both computationally and experimentally for the concentration used in the present work and that such a phenomenon could contribute to the change in viscosity observed in QCM-D experiments. A higher affinity of caffeine for the water solution can also explain the low encapsulation efficiency reported for liposomes in earlier studies.^20,22−24^

**Figure 5:**
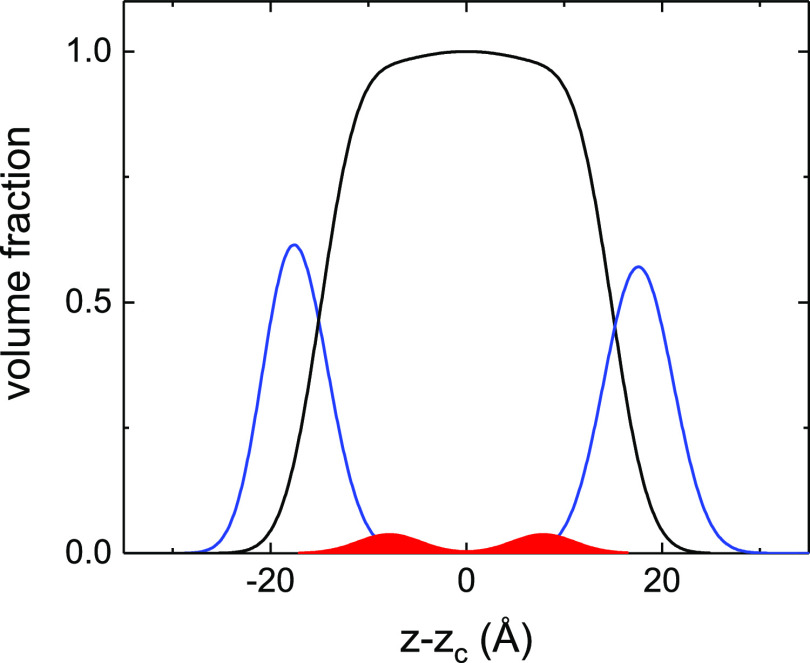
Volume fraction profiles of the organic components reconstructed from independent modeling of the NR data performed using a component group representation. The *z*-axis was shifted to center the profile of the bilayer at *z* – *z*_c_ = 0. Solid lines indicate the volume fraction distributions of the phospholipid molecules (black: acyl chains; blue: headgroups) while the red areas indicate the distribution of caffeine molecules.

Caffeine-containing SLBs were prepared from lipid films formed in the presence of caffeine. NR experiments identified the average position of caffeine molecules as in the hydrophobic portion of the bilayer. This conclusion was further supported by an additional analysis of NR data involving the use of a component group description similar to that previously published by some of us.^55^ The individual volume fraction contributions of headgroups, tails, and caffeine molecules were described by distributions composed by two symmetric error function profiles connected by a central step function of variable width. Within the bilayer regions, lipid, caffeine, and water contributions sum up to one by definition.^55^ The corresponding SLD profile can then be obtained by the sum of the individual volume fraction profiles once multiplied by the nominal SLD value of the corresponding molecular groups. This modeling approach is independent of the slab model used to determine the parameters shown in Table 1, and it was used solely to visualize the location of caffeine molecules as shown in Figure 5, where the volume fraction distribution of caffeine molecules is plotted against those of the hydrophobic and hydrophilic portions of the bilayer. This comparison provides evidence that caffeine is located entirely within the acyl chains region. In the presence of caffeine, we also observed a small decrease in total bilayer thickness, probably originating from the modified packing of phospholipid molecules. These findings are in contradiction with the conclusion derived by Khondker and co-workers^28^ from XRD experiments and MD simulations. It is worth mentioning that the systems investigated in the two cases were not exactly equivalent despite their very similar compositions. Khondker and co-workers investigated a solid-supported multilamellar stack consisting of approximately 3000 highly oriented bilayers. The sample was then measured against a humid environment at a maximum relative humidity of 93%. In the present work, the samples consisted of a single solid-supported bilayer immersed in water. These differences in experimental conditions might therefore explain the different locations of caffeine and the modification of lipid packing. Single SLBs are usually characterized by a larger mobility than that of a bilayer in a stack. In addition, bilayer stacks are usually prepared by evaporation of an organic solvent,^56^ and therefore the systems are very poorly hydrated during self-assembly. More importantly, molecular trafficking and exchange between the bilayer and the surrounding aqueous environment is enabled, while it is hindered in multilamellar bilayer stacks. This gives to SLBs a larger possibility of a molecular reorganization in response to the structural perturbation induced by the presence of caffeine molecules. Such a reorganization might allow caffeine molecules to migrate deeper into the hydrophobic portion of the bilayer. It is also possible that such a rearrangement took place already within the vesicles before the formation of the SLB. In agreement with the results already published in the literature,^28^ our analysis clearly indicate an increase in hydration of the headgroup region for the samples containing caffeine. This was confirmed for all of the samples despite their isotopic composition. This increase can be explained assuming that caffeine molecules are inserted with their longer axis (disk-like shape, ∼10 Å long) parallel to the acyl chains, promoting an increase in the average area per molecule, of the headgroup hydration and a decrease of the total bilayer thickness. Despite the low caffeine volume fraction and the relatively small perturbations caused to the pristine SLB structure, all of the models tested indicate the above-described scenario as the most likely. In particular, they indicated that the differences in reflectivity visible in Figure 2 originated from an increase of the tail SLD values in all contrast conditions. This observation is in line with the presence of caffeine in the hydrophobic region of the bilayer and rules out other scenarios as described in the Supporting Information material (Section 1.3).

## Conclusions

We investigated the interaction occurring between caffeine and a single supported lipid bilayer in the fluid phase composed by POPC. By combining quartz–crystal microbalance and neutron reflectometry techniques, we determined that, if originally present, caffeine molecules lay parallel to the acyl chains in the hydrophobic region of a bilayer. Because of the inclusion of caffeine molecules, the volume available for each phospholipid molecule increases, and this leads in turn to a larger headgroup hydration and a decrease in the total bilayer thickness. If added to a water solution, even at a concentration very close to the solubility limit, caffeine molecules do not partition into a bilayer environment. This is in line with earlier reports on the low encapsulation of caffeine in liposomes.^20,22−24^ Overall, these findings provide a novel point of view to improve the delivery of caffeine via membrane permeation and for its use in drug formulations and dietary supplements.

## Supporting Information Available

Molecular constraints used for the analysis of NR data; additional models tested during model assessment; NR curves and analysis for the *d*_31_POPC + caffeine sample; and NR data measured before, during, and after incubation of a POPC SLB with a 0.1 mol/kg caffeine solution (PDF)

## References

[ref1] Tavagnacco L.; Brady J.; Bruni F.; Callear S.; Ricci M.; Saboungi M.; Cesàro A. Hydration of caffeine at high temperature by neutron scattering and simulation studies. J. Phys. Chem. B 2015, 119, 13294–13301. 10.1021/acs.jpcb.5b09204. 26421842

[ref2] Eimer W.; Dorfmueller T. Self-aggregation of guanosine 5′-monophosphate, studied by dynamic light scattering techniques. J. Phys. Chem. A 1992, 96, 6790–6800. 10.1021/j100195a048.

[ref3] Chandler D. Interfaces and the driving force of hydrophobic. Nature 2005, 640–647. 10.1038/nature04162. 16193038

[ref4] Tavagnacco L.; Zaccarelli E.; Chiessi E. Molecular description of the coil-to-globule transition of Poly(N-isopropylacrylamide) in water/ethanol mixture at low alcohol concentration. J. Mol. Liq. 2020, 297, 111928 10.1016/j.molliq.2019.111928.

[ref5] Tavagnacco L.; Schnupf U.; Mason P. E.; Saboungi M.-L.; Cesàro A.; Brady J. W. Molecular dynamics simulation studies of caffeine aggregation in aqueous solution. J. Phys. Chem. B 2011, 115, 10957–10966. 10.1021/jp2021352. 21812485 PMC3189405

[ref6] Tavagnacco L.; Di Fonzo S.; D’Amico F.; Masciovecchio C.; Brady J.; Cesàro A. Stacking of purines in water: the role of dipolar interactions in caffeine. Phys. Chem. Chem. Phys. 2016, 18, 13478–13486. 10.1039/C5CP07326J. 27127808

[ref7] Tavagnacco L.; Gerelli Y.; Cesàro A.; Brady J. W. Stacking and branching in self-aggregation of caffeine in aqueous solution: from the supramolecular to atomic scale clustering. J. Phys. Chem. B 2016, 120, 9987–9996. 10.1021/acs.jpcb.6b06980. 27579545

[ref8] Yakovchuk P.; Protozanova E.; Frank-Kamenetskii M. D. Base-stacking and base-pairing contributions into thermal stability of the DNA double helix. Nucleic Acids Res. 2006, 34, 564–574. 10.1093/nar/gkj454. 16449200 PMC1360284

[ref9] Di Fonzo S.; Bottari C.; Brady J. W.; Tavagnacco L.; Caterino M.; Petraccone L.; Amato J.; Giancola C.; Cesàro A. Crowding and conformation interplay on human DNA G-quadruplex by ultraviolet resonant Raman scattering. Phys. Chem. Chem. Phys. 2019, 21, 2093–2101. 10.1039/C8CP04728F. 30638221

[ref10] Tavagnacco L.; Engström O.; Schnupf U.; Saboungi M.-L.; Himmel M.; Widmalm G.; Cesàro A.; Brady J. W. Caffeine and sugars interact in aqueous solutions: a simulation and NMR study. J. Phys. Chem. B 2012, 116, 11701–11711. 10.1021/jp303910u. 22897449 PMC3477616

[ref11] Tavagnacco L.; Brady J. W.; Cesàro A. The Interaction of sorbitol with caffeine in aqueous solution. Food Biophys. 2013, 8, 216–222. 10.1007/s11483-013-9290-7. 24000279 PMC3758476

[ref12] Shumilin I.; Allolio C.; Harries D. How sugars modify caffeine self-association and solubility: resolving a mechanism of selective hydrotropy. J. Am. Chem. Soc. 2019, 141, 18056–18063. 10.1021/jacs.9b07056. 31619038

[ref13] Brady J. W.; Tavagnacco L.; Ehrlich L.; Chen M.; Schnupf U.; Himmel M. E.; Saboungi M.-L.; Cesàro A. Weakly hydrated surfaces and the binding interactions of small biological solutes. Eur. Biophys. J. 2012, 41, 369–377. 10.1007/s00249-011-0776-2. 22124617

[ref14] Vraneš M.; Panić J.; Tot A.; Gadžurić S.; Podlipnik Č.; Bešter-Rogač M. How the presence of ATP affect caffeine hydration and self-aggregation?. J. Mol. Liq. 2020, 318, 113885 10.1016/j.molliq.2020.113885.

[ref15] Sharma B.; Paul S. Action of caffeine as an amyloid inhibitor in the aggregation of aβ16-22 peptides. J. Phys. Chem. B 2016, 120, 9019–9033. 10.1021/acs.jpcb.6b03892. 27487451

[ref16] Chrzanowska M.; Kuehn M.; Hermann T.; Neubert R. H. H. Biopharmaceutical characterization of some synthetic purine drugs. Pharmazie 2003, 58, 504–506. 12889537

[ref17] Carrillo J. A.; Benitez J. Clinically significant pharmacokinetic interactions between dietary caffeine and medications. Clin. Pharmacokinet. 2000, 39, 127–153. 10.2165/00003088-200039020-00004. 10976659

[ref18] Laska E. M.; Sunshine A.; Zighelboim I.; Roure C.; Marrero I.; Wanderung J.; Olson N. Effect of caffeine on acetaminophen analgesia. Clin. Pharmacol. Ther. 1983, 33, 498–509. 10.1038/clpt.1983.68. 6831829

[ref19] Derry C.; Derry S.; Moore R. Caffeine as an analgesic adjuvant for acute pain in adults. Cochrane Database Syst. Rev. 2014, CD009281 10.1002/14651858.CD009281.pub3. 25502052 PMC6485702

[ref20] Chorilli M.; Calixto G.; Rimério T. C.; Scarpa M. V. Caffeine encapsulated in small unilamellar liposomes: characerization and in vitro release profile. J. Dispersion Sci. Technol. 2013, 34, 1465–1470. 10.1080/01932691.2012.739535.

[ref21] Byun S. Y.; Kwon S. H.; Heo S. H.; Shim J. S.; Du M. H.; Na J. I. Efficacy of slimming cream containing 3.5% water-soluble caffeine and xanthenes for the treatment of cellulite: Clinical study and literature review. Ann. Dermatol. 2015, 27, 243–249. 10.5021/ad.2015.27.3.243. 26082579 PMC4466275

[ref22] Abd E.; Roberts M. S.; Grice J. E. A comparison of the penetration and permeation of caffeine into and through human epidermis after application in various vesicle formulations. Skin Pharmacol. Physiol. 2016, 29, 24–30. 10.1159/000441040. 26540487

[ref23] Tuncay Tanriverdi S. Preparation and characterization of caffeine loaded liposome and ethosome formulations for transungual application. Turk. J. Pharm. Sci. 2018, 15, 178–183. 10.4274/tjps.22931. 32454658 PMC7228016

[ref24] Kim C.; Shim J.; Han S.; Chang I. The skin-permeation-enhancing effect of phosphatidylcholine: caffeine as a model active ingredient. J. Cosmet. Sci. 2002, 53, 363–374. 12512013

[ref25] Ramsden J. J. Partition coefficients of drugs in bilayer lipid membranes. Experientia 1993, 49, 688–692. 10.1007/BF01923952. 8359275

[ref26] Budai L.; Kaszás N.; Gróf P.; Lenti K.; Maghami K.; Antal I.; Klebovich I.; Petrikovics I.; Budai M. Liposomes for topical use: A physico-chemical comparison of vesicles prepared from egg or soy lecithin. Sci. Pharm. 2013, 81, 1151–1166. 10.3797/scipharm.1305-11. 24482779 PMC3867246

[ref27] Paloncýová M.; Devane R.; Murch B.; Berka K.; Otyepka M. Amphiphilic drug-like molecules accumulate in a membrane below the head group region. J. Phys. Chem. B 2014, 118, 1030–1039. 10.1021/jp4112052. 24417480

[ref28] Khondker A.; Dhaliwal A.; Alsop R. J.; Tang J.; Backholm M.; Shi A.-C. C.; Rheinstädter M. C. Partitioning of caffeine in lipid bilayers reduces membrane fluidity and increases membrane thickness. Phys. Chem. Chem. Phys. 2017, 19, 7101–7111. 10.1039/C6CP08104E. 28229140

[ref29] Klamt A.; Huniar U.; Spycher S.; Keldenich J. COSMOmic: A mechanistic approach to the calculation of membrane-water partition coefficients and internal distributions within membranes and micelles. J. Phys. Chem. B 2008, 112, 12148–12157. 10.1021/jp801736k. 18754634

[ref30] Kalb E.; Frey S.; Tamm L. K. Formation of supported planar bilayers by fusion of vesicles to supported phospholipid monolayers. Biochim. Biophys. Acta, Biomembr. 1992, 1103, 307–316. 10.1016/0005-2736(92)90101-Q. 1311950

[ref31] Tavagnacco L.; Mason P. E.; Neilson G. W.; Saboungi M. L.; Cesàro A.; Brady J. W. Molecular dynamics and neutron scattering studies of mixed solutions of caffeine and pyridine in water. J. Phys. Chem. B 2018, 122, 5308–5315. 10.1021/acs.jpcb.7b07798. 29092394

[ref32] Sauerbrey G. Verwendung von schwingquarzen zur wägung dünner schichten und zur mikrowägung. Z. Phys. 1959, 155, 206–222. 10.1007/BF01337937.

[ref33] Höök F.; Rodahl M.; Brzezinski P.; Kasemo B. Energy dissipation kinetics for protein and antibody-antigen adsorption under shear oscillation on a quartz crystal microbalance. Langmuir 1998, 14, 729–734. 10.1021/la970815u.

[ref34] Montis C.; Gerelli Y.; Fragneto G.; Nylander T.; Baglioni P.; Berti D. Nucleolipid bilayers: A quartz crystal microbalance and neutron reflectometry study. Colloids Surf., B 2016, 137, 203–213. 10.1016/j.colsurfb.2015.07.039. 26233685

[ref35] Saerbeck T.; Cubitt R.; Wildes A.; Manzin G.; Andersen K. H.; Gutfreund P. Recent upgrades of the neutron reflectometer D17 at ILL. J. Appl. Crystallogr. 2018, 51, 249–256. 10.1107/S160057671800239X.

[ref36] Daillant J.; Gibaud A. X-ray and Neutron Reflectivity; Daillant J.; Gibaud A., Eds.; Lecture Notes in Physics; Springer Berlin Heidelberg: Berlin, Heidelberg, 2009; Vol. 770, p 331.

[ref37] Crowley T. L.; Lee E. M.; Simister E. A.; Thomas R. K. The use of contrast variation in the specular reflection of neutrons from interfaces. Phys. B 1991, 173, 143–156. 10.1016/0921-4526(91)90044-F.

[ref38] Gerelli Y. Aurore: new software for neutron reflectivity data analysis. J. Appl. Crystallogr. 2016, 49, 330–339. 10.1107/S1600576716000108.

[ref39] Harroun T. A.; Kučerka N.; Nieh M.-P.; Katsaras J. Neutron and X-ray scattering for biophysics and biotechnology: examples of self-assembled lipid systems. Soft Matter 2009, 5, 2694 10.1039/b819799g.

[ref40] Fragneto G. Neutrons and model membranes. Eur. Phys. J.: Spec. Top. 2012, 213, 327–342. 10.1140/epjst/e2012-01680-5.

[ref41] Junghans A.; Watkins E. B.; Barker R. D.; Singh S.; Waltman M. J.; Smith H. L.; Pocivavsek L.; Majewski J. Analysis of biosurfaces by neutron reflectometry: from simple to complex interfaces. Biointerphases 2015, 10, 019014 10.1116/1.4914948. 25779088

[ref42] Welbourn R.; Clarke S. New insights into the solid-liquid interface exploiting neutron reflectivity. Curr. Opin. Colloid Interface Sci. 2019, 42, 87–98. 10.1016/j.cocis.2019.03.007.

[ref43] Gerelli Y. Applications of neutron reflectometry in biology. EPJ Web Conf. 2020, 236, 04002 10.1051/epjconf/202023604002.

[ref44] Parratt L. G. Surface studies of solids by total reflection of X-rays. Phys. Rev. 1954, 95, 359–369. 10.1103/PhysRev.95.359.

[ref45] James F. MINUIT Function Minimization and Error Analysis, 1998.

[ref46] Gerelli Y. Phase transitions in a single supported phospholipid bilayer: real-time determination by neutron reflectometry. Phys. Rev. Lett. 2019, 122, 248101 10.1103/PhysRevLett.122.248101. 31322398

[ref47] Gerelli Y.; Eriksson Skog A.; Jephthah S.; Welbourn R. J. L.; Klechikov A.; Skepö M. Spontaneous formation of cushioned model membranes promoted by an intrinsically disordered protein. Langmuir 2020, 36, 3997–4004. 10.1021/acs.langmuir.0c00120. 32212610 PMC7311080

[ref48] Kučerka N.; Nieh M.-P.; Katsaras J. Fluid phase lipid areas and bilayer thicknesses of commonly used phosphatidylcholines as a function of temperature. Biochim. Biophys. Acta, Biomembr. 2011, 1808, 2761–2771. 10.1016/j.bbamem.2011.07.022. 21819968

[ref49] Shen H. H.; Hartley P. G.; James M.; Nelson A.; Defendi H.; McLean K. M. The interaction of cubosomes with supported phospholipid bilayers using neutron reflectometry and QCM-D. Soft Matter 2011, 7, 8041–8049. 10.1039/c1sm05287j.

[ref50] Fogarty J. C.; Arjunwadkar M.; Pandit S. A.; Pan J. Atomically detailed lipid bilayer models for the interpretation of small angle neutron and X-ray scattering data. Biochim. Biophys. Acta, Biomembr. 2015, 1848, 662–672. 10.1016/j.bbamem.2014.10.041. 25448879

[ref51] Montis C.; Salvatore A.; Valle F.; Paolini L.; Carlà F.; Bergese P.; Berti D. Biogenic supported lipid bilayers as a tool to investigate nano-bio interfaces. J. Colloid Interface Sci. 2020, 570, 340–349. 10.1016/j.jcis.2020.03.014. 32171928

[ref52] Greenwood A. I.; Tristram-Nagle S.; Nagle J. F. Partial molecular volumes of lipids and cholesterol. Chem. Phys. Lipids 2006, 143, 1–10. 10.1016/j.chemphyslip.2006.04.002. 16737691 PMC2695672

[ref53] Bahrami H.; Tabrizchi M.; Farrokhpour H. Protonation of caffeine: a theoretical and experimental study. Chem. Phys. 2013, 415, 222–227. 10.1016/j.chemphys.2013.01.022.

[ref54] Kučerka N.; Tristram-Nagle S.; Nagle J. F. Structure of fully hydrated fluid phase lipid bilayers with monounsaturated chains. J. Membr. Biol. 2006, 208, 193–202. 10.1007/s00232-005-7006-8. 16604469

[ref55] Belička M.; Gerelli Y.; Kučerka N.; Fragneto G. The component group structure of DPPC bilayers obtained by specular neutron reflectometry. Soft Matter 2015, 11, 6275–6283. 10.1039/C5SM00274E. 26160133

[ref56] Tristram-Nagle S. A. Methods in Molecular Biology; Humana Press: New Jersey, 2007; Vol. 400, pp 63–75. 17951727 10.1007/978-1-59745-519-0_5 PMC2697614

