# Supplementary material for: Interaction of Caffeine with Model Lipid Membranes
Source: J Phys Chem B. 2021 Sep 1;125(36):10174–81. doi: 10.1021/acs.jpcb.1c04360 (PMC8450902; doi:10.1021/acs.jpcb.1c04360)
Supplement: Supplementary file 1 — jp1c04360_si_001.pdf [file jp1c04360_si_001.pdf]

Supporting Information: Interaction of Caffeine with Model Lipid Membranes

Letizia Tavagnacco,† Giacomo Corucci,‡ and Yuri Gerelli*,¶

†*CNR-ISC and Department of Physics, Sapienza University of Rome, Piazzale A. Moro 2, 00185, Rome, Italy*

‡*Institut Laue-Langevin, 71 avenue des Martyrs, 38000, Grenoble, France*

¶*Department of Life and Environmental Sciences, Marche Polytechnic University, Via Brecce Bianche, 60121, Ancona, Italy*

E-mail: y.gerelli@univpm.it

1 Details on the analysis of NR data

1.1 SLBs without caffeine

The analysis of caffeine-free SLBs was performed by applying the molecular constraints described for the *lipid plug-in* of the Aurore software application.[S1] Within this framework, SLBs were modeled as symmetric, with structural parameters shared among the two leaflets that were assumed to be characterized by the same interfacial roughness value (σ).

By using the molecular information available, the number of free parameters was reduced by applying the following constraints:

$$\begin{aligned}
\rho_H^{dry} &= \frac{b_H}{V_H^{dry}} & \rho_C^{dry} &= \frac{b_C}{V_C^{dry}} \\
V_H &= (\mathbf{n_H^w} V_w + V_H^{dry}) & V_C &= (\mathbf{n_C^w} V_w + V_C^{dry}) \\
f_H &= \frac{\mathbf{n_H^w} V_w}{V_H} & f_C &= \frac{\mathbf{n_C^w} V_w}{V_C} \\
A_{lip} &= \frac{V_H}{\mathbf{t_H}} & t_C &= \frac{V_C}{A_{lip}} \\
V_C^{dry} &= V_{lip} - V_H^{dry}
\end{aligned} \tag{S1}$$

In the relations described above, b_H and b_C are the sum of the coherent scattering lengths of all the nuclei contained in the volumes V_H^{dry} and V_C^{dry} respectively. The index *dry* indicates that these calculations were made without including any water molecule at this stage. These values, taken from the literature, were kept fixed during the minimization. Presence of water in the tail and headgroup regions was accounted for by using the water volume fractions parameters f_H and f_C as

$$\rho_x = f_x \rho_w + (1 - f_x)\rho_x^{dry} \tag{S2}$$

where x is a stand-in index for C and H and ρ_w is the SLD of the aqueous phase. Other symbols not described in the main manuscript indicate the number of water molecules present in the head (n_H^w) and tails (n_C^w) regions, the volume of a water molecule (V_w, fixed to 30 Å^3),

the area/lipid A_{lip} and the total lipid molecular volume V_{lip}. For a symmetric lipid bilayer the total number of parameters was reduced to 4, namely t_H, σ, n_H^w and n_C^w. They are in bold in the dependencies listed in S1.

The free parameters in S1 were varied within the following parameter bounds:

$$\begin{aligned} 5\,\text{Å} \;&\leq\; t_H \;\leq\; 12\,\text{Å} \\ 0 \;&\leq\; n_H^w \;\leq\; 20 \\ 0 \;&\leq\; n_C^w \;\leq\; 10 \\ 2\,\text{Å} \;&\leq\; \sigma \;\leq\; 10\,\text{Å} \end{aligned} \tag{S3}$$

1.2 SLBs with caffeine

Given the low volume fraction of caffeine used, the modeling of NR data of SLBs in the presence of caffeine was performed assuming that the presence of caffeine would result in a small perturbation of the pristine SLB structure. The corresponding NR data were therefore analysed using as starting parameters those of pure SLBs. Moreover, since caffeine was present in the vesicles used to form SLBs, the bilayer structure was considered to remain symmetric (i.e. the two leaflets shared a common set of parameters). Alternative modeling approaches (including one assuming asymmetric leaflets) were tested as described in Section 1.3 and resulted not applicable. In this *small perturbation* approach the following parameters

were free to vary within the reported bounds:

$$\begin{aligned}
4\,\text{Å} &\le t_H \le 12\,\text{Å} \\
1.7\times10^{-6}\,\text{Å}^{-2} &\le \rho_H \le 4.0\times10^{-6}\,\text{Å}^{-2} \\
0 &\le f_H \le 1 \\
12\,\text{Å} &\le t_C \le 20\,\text{Å} \\
-0.29\times10^{-6}\,\text{Å}^{-2} &\le \rho_C \le 2.0\times10^{-6}\,\text{Å}^{-2} \\
0 &\le f_C \le 1 \\
2\,\text{Å} &\le \sigma \le 7\,\text{Å}
\end{aligned} \tag{S4}$$

The roughness parameter σ was constrained to a lower superior bound because in some cases it could reach values as large as 9 Å. Such a large value would affect the correct interpretation of the modeling, resulting in smearing effect on the thinner headgroup region and into a non physical split of the SLD profiles at the center of the bilayer. Constraining this parameter to values smaller than 7 Å resulted in a better reproducibility of the analysis and agreement with the results already reported in literature for similar caffeine concentrations in lipid bilayers.

1.3 Alternative modeling approaches

While evaluating the goodness of the modeling approach, the symmetric bilayer model with caffeine molecules located among the tails was tested against other *a priori* possible configurations including:

1. caffeine in both tails and headgroup regions (symmetric)

2. caffeine in headgroup regions only (symmetric)

3. caffeine in one leaflet only (asymmetric)

4. caffeine in the center of the bilayer (among methyl groups)

5. absence of caffeine and SLB low coverage

Model 1 was the final model chosen for the analysis of the data, in which the SLD of the headgroups remained equal to that of a SLB in the absence of caffeine. In the best case scenario the likelihood parameter χ^2 resulted 2.96.

Model 2 was tested by keeping fixed $\rho_C = -0.29 \times 10^{-6}$ Å^{-2} and $t_C = 15.0$ Å while allowing all the other parameters to vary within the limits reported in S4. In the best case scenario the likelihood parameter χ^2 resulted 6.56. Moreover, the ρ_H value did not change, indicating no evidence of caffeine partitioning among the headgroups.

Model 3 was tested by fixing the parameters of one leaflet to those found for the SLB in the absence of caffeine while keeping those of the opposite leaflet free to vary within the bounds reported in S4. Depending on which leaflet was assumed to contain caffeine, the likelihood parameter χ^2 resulted 5.55 (caffeine in the inner leaflet) or 5.73 (caffeine in the outer leaflet). In both cases the curves reproduced poorly the experimental data.

Model 4 was tested by adding a layer in the center of the bilayer. Its thickness could vary between 0 Å and 10 Å the *dry* SLD was fixed to that of caffeine (3.3 $\times 10^{-6}$ Å^{-2}), the water volume fraction could vary between 0 and 1 and the roughness was described by σ. In all the runs of the minimization procedure, carried out either by fixing or letting free to vary the headgroup and tail parameters, the thickness of this layer was found to be 0.0 Å. If thickness was force to be larger than 2 Å then the likelihood parameter χ^2 resulted 17.64. The optimal model, with a 0 thickness, corresponded to the optimal structure found for model 1.

Model 5 was tested by using the molecular constraints reported in S1 with the parameters free to vary as described in S3. This model systematically failed because changes in the reflectivity curves in the presence of caffeine could not be described in terms of larger water volume fraction in the tails and headgroup regions. As reported in the main manuscript, these changes were originated by the inclusion of material with a positive SLD, which is not compatible with the presence of H_2O since it has a negative SLD. In the best case scenario

the likelihood parameter χ^2 resulted 8.61.

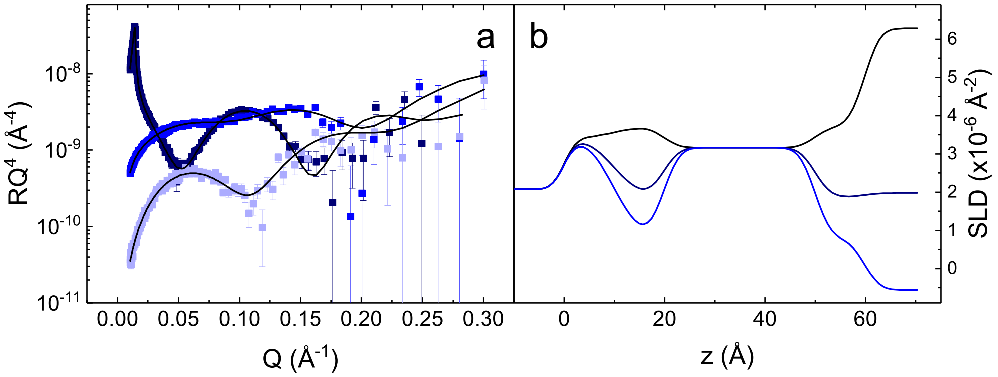

Figure **S1**: (**a**) NR curves (symbols) for the SLB containing POPC and caffeine (at 5 mol%) in D_2O (dark blue), SiMW (light blue) and H_2O (blue). Solid lines represent the results obtained by a global fit of the NR curves shown. (**b**) SLD profiles corresponding to the model fits reported in panel (a) (the same color code applies).

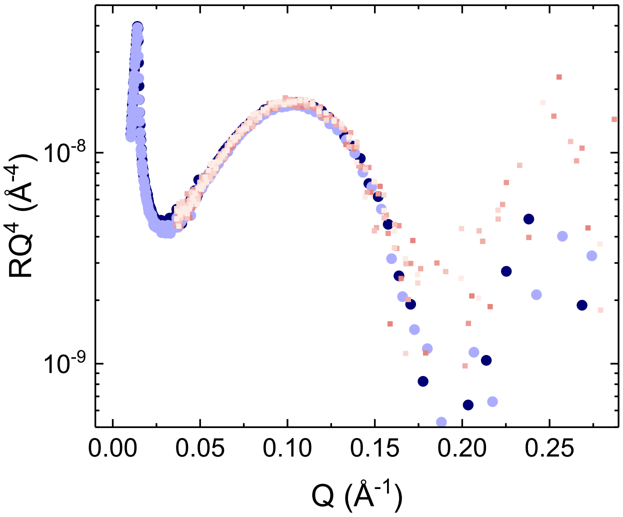

Figure **S2**: NR data for a POPC SLB in D_2O before (dark blue circles) and after (light blue circles) in incubation with 0.1 *m* caffeine solution. Time resolved measurements collected on a restricted Q-range are plotted as squares (shades of red). Error-bars were removed for clarity. No differences outside of the experimental accuracy were observed.

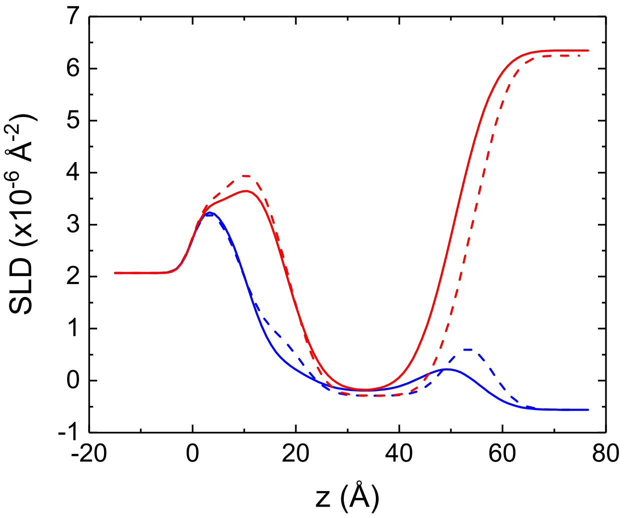

Figure **S3**: Comparison between the SLD profiles for a POPC SLB (dashed lines) and for a POPC SLB pre-loaded with caffeine (solid lines) in D_2O (red) and in H_2O (blue). The structural parameters describing these profiles are given in Table 1 in the main manuscript. Visual differences in the hydrophilic regions of the bilayer ($10<z<20$ Å and $50<z<60$ Å) are induced by the larger hydration of the POPC+caffeine sample.

References

(S1) Gerelli, Y. Aurore : new software for neutron reflectivity data analysis. *J. Appl. Crystallogr.* **2016**, *49*, 330–339.
